# Geothermal Nano-SiO_2_ Waste as a Supplementary Cementitious Material for Concrete Exposed at High Critical Temperatures

**DOI:** 10.3390/ma17174381

**Published:** 2024-09-05

**Authors:** Jesús Fernando López-Perales, María Cruz Alonso-Alonso, Francisco Javier Vázquez-Rodríguez, Ana María Guzmán-Hernández, Lauren Yolanda Gómez-Zamorano, Edén Amaral Rodríguez-Castellanos, Rodrigo Puente-Ornelas

**Affiliations:** 1 Facultad de Ingeniería Mecánica y Eléctrica, Universidad Autónoma de Nuevo León, Av. Pedro de Alba s/n, Ciudad Universitaria, San Nicolás de los Garza C.P. 66450, Nuevo Leon, Mexico; jlopezp@uanl.edu.mx (J.F.L.-P.); ana.guzmanhr@uanl.edu.mx (A.M.G.-H.); lauren.gomezzm@uanl.edu.mx (L.Y.G.-Z.); 2 Consejo Superior de Investigaciones Científicas, Instituto de Ciencias de la Construcción Eduardo Torroja, C/Serrano Galvache 4, 28033 Madrid, Spain; mcalonso@ietcc.csi.es; 3 Programa Doctoral en Ingeniería Física, Facultad de Ciencias Físico Matemáticas, Universidad Autónoma de Nuevo León, Av. Pedro de Alba s/n, Ciudad Universitaria, San Nicolás de los Garza C.P. 66451, Nuevo Leon, Mexico; fcofimeuanl@gmail.com; 4 Facultad de Arquitectura, Universidad Autónoma de Nuevo León, Av. Pedro de Alba s/n, Ciudad Universitaria, San Nicolás de los Garza C.P. 66451, Nuevo Leon, Mexico

**Keywords:** supplementary cementitious material (SCM), geothermal nano-SiO_2_ waste (GNSW), ultrasonic pulse velocity (UPV), composite Portland cement (CPC), thermogravimetric analysis (TG)

## Abstract

The partial replacement effect of Portland cement by geothermal nano-SiO_2_ waste (GNSW) for sustainable Portland-cement-based concrete was investigated to improve the properties of concrete exposed at high critical temperatures. Portland cement was partially replaced by 20 and 30 wt.% of GNSW. The partial replacement effect on Portland-cement-based concrete subjected to 350, 550, and 750 °C was evaluated by measuring the weight changes, ultrasonic pulse velocity, thermogravimetric and differential thermal analysis, X-ray diffraction, surface inspection, and scanning electron microscopy under residual conditions. The ultrasonic pulse velocity results showed that the GNSW specimens maintained suitable stability after being heated to 350 °C. The SEM analysis revealed a denser microstructure for the 20 wt.% of partial replacement of Portland cement by GNSW specimen compared to the reference concrete when exposed to temperatures up to 400 °C, maintaining stability in its microstructure. The weight losses were higher for the specimens with partial replacements of GNSW than the reference concrete at 550 °C, which can be attributed to the pozzolanic activity presented by the GNSW, which increases the amounts of CSH gel, leading to a much denser cementitious matrix, causing a higher weight loss compared to the reference concrete. GNSW is a viable supplementary cementitious material, enhancing thermal properties up to 400 °C due to its high pozzolanic activity and filler effect while offering environmental benefits by reducing industrial waste.

## 1. Introduction

Portland cement concrete is the most predominant construction material worldwide due to its durability, resistance, chemical stability, reduced production cost, and easy shaping ability [1,2]. Today, the demand for concrete is ascendant, and no other building material can equal its attractive properties [3,4]. Nevertheless, the environmental footprint has been expanded since the Portland cement (PC) making process causes greenhouse gas emissions such as CO_2_, SO_x_, and NO_x_ due to the calcination of raw materials at temperatures above 1450 °C [5]. CO_2_ emissions associated with cement production (around 7–9%) demand new environmentally friendly solutions that reduce the carbon footprint [1,6,7,8]. Modernization in Portland-cement-making processes has emerged, reaching improved properties, reducing the environmental footprint from the production process, and reducing the PC production cost [9]. Moreover, one popular initiative is composite cement use, whose composition is modified by a proportion of Portland cement replacement by industrial by-products with high production volumes [10,11,12], such as pozzolanic and hydraulic materials [13,14]. These cements show attractive properties such as delaying or reducing heat of hydration, improved durability, and developing higher resistance than those indicated by the standards [15,16]. Alternative materials used as a partial or total replacement of PC represent an attractive advantage in the building industry with outstanding results and a promising future, not only as an ecological solution but also as a technological implement due to its improved properties [14]. Hence, it is crucial to seek alternative materials as supplementary cementitious materials (SCMs) in nature to expand its chances in the building industry.

As reported, supplementary cementitious materials (SCMs) in concrete mixtures as a partial replacement for Portland cement have become a regular practice in the construction industry. These technological implementations provide more environmental advantages to the concrete technology besides mechanical resistance increase and durability to the concrete structures [15,17,18,19]. The main reasons why SCMs are extensively used in concrete mixtures are based on three concepts: (i) a need to reduce the environmental impact of Portland cement manufacture, as this process requires energy consumption in large quantities, which emits greenhouse gases in similar quantities, mainly CO_2_; (ii) the sustainability of the cement industry since it has been threatened by the rising price of fossil fuels and eco-taxes for the release of CO_2_; and (iii) the improvement of concrete properties. Nowadays, SCMs include industrial by-products such as fly ash (FA), granulated blast furnace slag (GBFFS), silica fume (SF), calcined clays, rice husk ash, and others in their manufacturing [20,21,22].

On the other hand, nanotechnological applications have gained popularity in different science and engineering fields, so this revolutionary concept has also favorably impacted construction materials. When nanoparticles have been used as SCMs, improvements have been achieved, such as their role as hydration nuclei, pozzolanic behavior, and their potential as a filler agent since they can fill the voids in the cement matrix [23,24]. In addition, the large surface area plus the small size of nanoparticles can facilitate the chemical reactions necessary to produce a dense cement matrix with more calcium silicate hydrate (C-S-H) and less calcium hydroxide content. The nanoparticles can also strengthen the interfacial transition zone between the cement paste and the aggregate, improving the strength and permeability [25,26].

Some works [27,28,29,30] that analyzed studies associating nano-silica particles in the construction field manifest their use alongside fly ash and other nanoparticles to improve the properties of concrete, such as compactness, strength, and durability. Adding silica nanoparticles to the concrete mix has a more pronounced effect than those obtained by adding silica microparticles. This behavior is mainly due to their surface area and a nucleation mechanism that promotes nucleation sites for CSH gel precipitation [31]. In cement pastes, it has been found that adding silica nanoparticles improves the thermal stability of the cementitious system [32]. Morteza et al. studied the effect of high temperatures on high-strength concrete with nano-silica additions of less than 5%, using a heating rate of 20 °C/min. The results showed that silica nanoparticles increase the compressive and residual tensile strengths of concrete specimens subjected to high temperatures more than silica fumes [31]. Ibrahim et al. studied high-strength mortars with fly ash additions in large volumes (25–45%) and nano-silica (2.5%, 5%, and 7.5%) exposed to temperatures up to 700 °C using a heating rate of 9 °C/min. The results showed that at 700 °C, the mortars exhibited a reduction in compressive and flexural strength; however, specimens containing nano-silica showed higher residual strength [33,34]. Heikal et al. studied cement composed of GBFFS and nano-silica in percentages up to 4%, where they were exposed to heat treatments at temperatures of 200, 400, 650, 850, and 900 °C at a heating rate of 5 °C/min. The results indicate that cement composed of GBFFS at 25% to 50% and nano-silica at percentages of 4% improves thermal resistance at temperatures up to 650 °C [23,26].

As observed, nano-silica particles as a pozzolanic material are preferred instead of silica fume at early ages due to a higher consumption rate of liberated Ca(OH)_2_ crystals from cement phase hydration. However, the drawback of nanomaterials applied to the construction industry is their price, considering that it is higher than the traditional pozzolanic materials. Therefore, the feasibility of using natural nanoparticles as a substitute for some SCMs must respond to a suitable cost/benefit ratio, i.e., an equal or better performance in combination with comparable or lower cost than the traditional SCMs [24].

Some research projects report geothermal silica (GS) use in cement technology [35,36,37]. GS is a by-product of the energy production process by steam extraction from the subsurface, which is received as a mixture of geothermal brine and steam that undergoes a series of steps to extract heat. It has been reported the high pozzolanic potential of GS leads to the densification of the Portland cement hydration product matrix [35]. Also, several studies have demonstrated the GS capability to be used in sustainable binders’ elaboration, such as alkali-activated (AA) binders [38,39,40,41,42,43,44,45,46,47,48,49,50]. Due to the presence of soluble silica and alumina in GS, the formation of reaction products develops new properties that contribute to the strength and stability of the Portland cement hydration product matrix [51,52]. GS has advantages over ordinary Portland cement concrete (OPC), such as rapid high-strength development, excellent durability, and high resistance to temperature and chemical attacks [39,43,51,53]. The benefits of using GS include reduced CO_2_ emissions and energy consumption associated with Portland cement production, as well as the preservation of non-renewable natural resources [1,2,39,40,54,55,56].

On the other hand, the Cerro Prieto geothermal plant (located in Baja California, Mexico) produces low-cost electricity. Some by-products corresponding to amorphous silica nanoparticles (10–50 nm in size), sodium, and potassium chlorides are generated during the process. It is estimated that the waste generation is more than 70,000 tons per year. The GNSW is composed mainly of amorphous nano-silica, with sodium and potassium chlorides as minor elements further eliminated by a washing process. The literature indicates that GNSW has been used as a replacement of PC with and without other supplementary materials and in some cases with the aid of alkaline activation [36,57,58,59]. Hajimohammadi et al. [60] formulated geopolymers using GNSW and sodium aluminate powder. Escalante Garcia et al. [61,62] evaluated the addition of GNSW in alkali-activated slag binders and reported an increase in the reactivity of the activated systems; enhanced strength was noted for activation with NaOH, while for activation with waterglass, the strength was reduced. Some studies have reported improvements in Portland-cement-based paste properties with the GNSW use as a partial replacement of Portland cement. This behavior was attributed to the pozzolanic activity of GNSW, which improved the hydration of the cement, increasing the amount of C-S-H gel and resulting in a more compact matrix of hydrated products [36,37,63]. The mechanical and durability properties of Portland-cement-based concrete with partial replacements of GNSW (up to 30 wt.%) show that the GNSW is a promising material as a replacement of Portland cement with benefits like those obtained with silica fume replacements but at less cost [64].

On the other hand, it is well known that during its service life, ordinary Portland cement concrete can be exposed accidentally or by design factors at high temperatures or fire, which causes a series of alterations in the chemical composition of cement paste and in the physical properties of aggregates. The high temperature causes mass loss, mechanical property decrement, and deterioration in durability, jeopardizing the concrete structure and sometimes human lives [65,66,67,68]. The damage that occurs in the concrete structures will mainly depend on the heating rate, the maximum temperature reached, the exposure time, the cooling conditions, and the material properties, which the concrete was made from, such as aggregates, cement type, further additions, water, and chemical additives [67,68].

Because of the relevance of concrete behavior at high temperatures and in fire, many studies have been carried out on cementitious composites at high temperatures, and the most relevant parameters have been identified and investigated [69,70,71]. Some authors have provided comprehensive and updated reports on the temperature dependency of parameters such as compressive strength, modulus of elasticity, strength in indirect tension (bending and splitting tests), stress–strain curves, and spalling [71,72]. 

Some efforts have been made to enhance the thermal stability of Portland-cement-based composites by incorporating supplementary cementitious materials. However, the properties of fire-damaged concrete should be studied further to improve the fire resistance of concrete containing by-products and supply significant data for the fire safety design of concrete structures [31,72,73,74,75,76,77].

Since previous research has found that geothermal nano-silica waste (GNSW) is a promising material as a partial replacement for Portland cement (up to 30 wt.%) and that it provides benefits such as mechanical property enhancement (compressive strength) and durability (electrochemical corrosion and accelerated carbonation) similar to those obtained with silica fume, the challenge of this research is to study the effect that the addition of GNSW has on the durability of concrete subjected to high temperatures to contribute knowledge in the development and improvement of alternative materials to Portland cement and, since it is an industrial residue, to contribute to the preservation of the environment.

## 2. Materials and Methods

### 2.1. Material Characterization

The concrete mixtures were elaborated using a compound Portland cement from CEMEX (Monterrey, Mexico), aggregate from mineral limestone (mountain deposits in Nuevo Leon, Mexico), distilled water, and superplasticizer Glenium 3150 polycarboxylate-based (BASF, Ludwigshafen, Germany). Commercial silica fume (SF) distributed by Degussa Mexico S.A. de C.V., Mexico was also used in this study.

The geothermal nano-silica waste (GNSW geothermal nano-SiO_2_) from the Cerro Prieto geothermal plant in Baja California, Mexico, was pretreated before being incorporated into the concrete mixture. The GNSW is a material containing sodium and potassium chlorides, which must be removed by washing, as they can have a negative effect on the behavior of the replaced cement pastes.

As known, the pretreatment of GNSW by the washing process has been documented with promising results, and the effect of the sodium and potassium chloride content present in GNSW has also been previously reported in mortars [58] and pastes [36,37,78,79,80,81]. The pretreatment consisted of a washing process to reduce sodium and potassium chloride content in the GNSW to approximately 0%. For this purpose, the treatment was carried out with potable water at ~100 °C, in a ratio of 3:1 water: GS, homogenizing it for 15 min. Subsequently, the mixture was left to decant for 12 h. Once the material had settled, the water containing the dissolved chloride was extracted, with the GNSW remaining. This washing process was carried out until the GNSW reached a concentration of total chlorides close to 0%. In this investigation, between each washing, a chloride analysis was carried out by titration. When the content was minimal (0.02%), the material was suitable to substitute the OPC [35]. After washing, the GNSW was held in a software-controlled isothermal Thermo Fisher Scientific chamber (Waltham, MA, USA) at a constant temperature of 60 °C. The waste remained in this environment for 48 h, where it was practically dried. Subsequently, the GNSW was deposited in a metal container and dried at 120 °C for 24 h in an electric furnace (Heratherm, Thermo Scientific, Waltham, MA, USA). 

As anticipated, the nanoparticles exhibit a tendency to agglomerate, which can adversely affect their performance in concrete mixtures. To achieve better dispersion and effectiveness, the geothermal nano-SiO_2_ waste underwent a process of deagglomeration. This process involved two key steps: pulverization and sieving. The pulverization process was carried out to reduce the particle size and break up agglomerates, improving the distribution of nanoparticles within the mixture. Subsequently, the material was sieved to achieve a consistent particle size distribution, with a targeted maximum particle size of 75 μm. This sieve size ensures that the nanoparticles are sufficiently fine to enhance their reactivity and performance while minimizing the impact of any remaining agglomerates. By controlling the particle size and distribution, the modified geothermal nano-SiO_2_ waste demonstrates improved behavior and effectiveness in concrete applications.

Pulverization process reduces the particle size of the nano-SiO_2_ waste, aiming to achieve a finer powder. Pulverization helps in breaking down agglomerates and decreasing the overall particle size. After pulverization, sieving through a mesh with a maximum aperture of 75 μm is employed. This ensures that the particle size does not exceed this limit, thus facilitating better dispersion in the concrete mix.

On the other hand, nano-sized particles naturally tend to clump together due to van der Waals forces. Effective deagglomeration is crucial for ensuring that these particles remain well-dispersed in the concrete mixture. Properly dispersed nanoparticles can improve the performance of concrete by enhancing hydration reactions and filling voids, which can contribute to better strength and durability. In addition, achieving the right particle size helps maintain the workability of the concrete mix, ensuring that it can be easily handled and placed. Finally, finer and well-dispersed nanoparticles contribute positively to the strength and durability of concrete by enhancing the formation of calcium silicate hydrate (C-S-H) gel.

**Figure 1:**
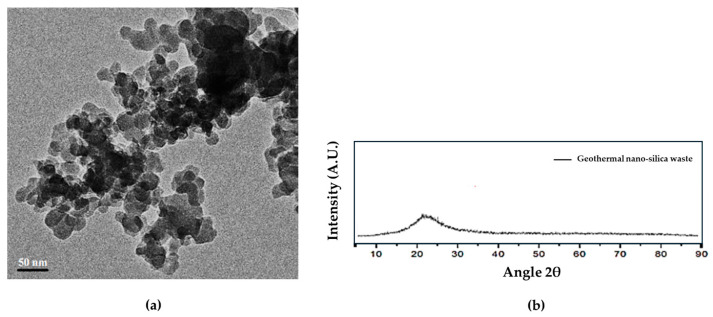
(**a**) Particle size of the GNSW measured by transmission electron microscopy and (**b**) X-ray diffraction pattern of GNSW showing the amorphous halo [78].

Figure 1a shows the particle size of the GNSW was measured by transmission electron microscopy (TEM-Jeol, JEM-2010 model, JEOL Ltd., Tokyo, Japan), and it was estimated to be in a range size of 10 to 50 nm [35]. 

The main characteristics of GNSW are published [13,15,82], showing only an amorphous halo between 20° and 30° 2θ. Figure 1b shows the X-ray diffraction pattern of GNSW.

Table 1 shows the chemical composition of the cement and the supplementary cementitious materials (determined by X-ray fluorescence), the density of the materials (determined by the Le Chaterlier method according to ASTM C-188), and the surface area, using the nitrogen absorption technique together with the BET analysis method (Bel Japan Inc.-Minisorp II model).

### 2.2. Mix Design

The water/cement ratio (w/c expressed by weight) was 0.5 in the elaboration of concrete specimens with a cement content of approximately 350 kg/m^3^. Concrete specimens were fabricated in a mixer with a capacity of 90 L using compound Portland cement (CPC 30R), geothermal nano-SiO_2_ waste (GNSW), silica fume (SF), coarse aggregate (19 mm), fine aggregate (4.75 mm) from mineral limestone, distilled water, and a polycarboxylate-based Glenium 3150 superplasticizer. 

Four experimental concretes were prepared. The C-100 concrete (reference concrete) consists of CPC, fine and coarse aggregates, and superplasticizers. The C-GNSW20 concrete corresponds to CPC, fine and coarse aggregates, superplasticizers, and 20 wt.% of partial replacement of compound Portland cement by geothermal nano-SiO_2_ waste. The C-GNSW30 concrete consists of CPC, fine and coarse aggregates, superplasticizers, and 30 wt.% of partial replacement of compound Portland cement by geothermal nano-SiO_2_ waste. The C-SF30 concrete as a comparative concrete consists of CPC, fine and coarse aggregates, superplasticizer, and 30 wt.% of partial replacement of compound Portland cement by silica fume. 

Replacing a significant amount of Portland cement with nano-sized materials such as geothermal nano-silica waste (GNSW) can be particularly challenging, and achieving such high replacement rates requires careful consideration of various factors.

Some studies have reported that nano-silica can replace up to 20–30% of cement while achieving desirable properties owing to its high reactivity and effective filling of voids [26,27,28,29,30,31]. Nano-sized particles have an extremely high surface area to volume ratio, which enhances their reactivity compared to conventional silica fume. This higher reactivity can contribute to better bonding with the cement matrix, allowing for higher replacement rates. Nano-sized materials often exhibit enhanced pozzolanic activity due to their large surface area, which leads to a more extensive reaction with calcium hydroxide. This fact can contribute to the formation of additional calcium silicate hydrate (C-S-H), improving the strength and durability of the concrete. Despite their small size, nano-sized materials can be more effective in contributing to the hydration reactions and filling the voids in the cement matrix, which helps achieve high replacement rates. The hydraulic reactivity of GNSW is crucial. Nano-sized particles can improve the pozzolanic reaction due to their high surface area since they react with calcium hydroxide (a by-product of cement hydration) more effectively than larger particles. GNSW, if it possesses high hydraulic reactivity, can serve as an effective supplementary cementitious material SCM, contributing to the strength and durability of the concrete. The higher the reactivity, the more effectively it can replace a significant portion of Portland cement. Adjustments in mix design are necessary to accommodate high replacement rates. These adjustments may include optimizing the water/cement ratio and incorporating chemical admixtures like superplasticizers to maintain workability.

Table 2 shows the designation (code) and mixture proportions of the concretes. After mixing, the fresh concrete was poured into cylindrical plastic molds (50 mm in diameter × 150 mm in height). Specimens were demolded after 24 h and placed in calcium hydroxide-saturated water to carry out a 365-day curing process according to the ASTM C31-18 standard. To simulate a condition where concrete may be exposed to high temperatures, a specimen batch was placed in a laboratory for an additional year of aging to allow complete hydration before being subjected to the heating regime.

The concrete properties at high temperatures were evaluated, using the most common way to study the influence of high temperature on the properties of concrete by exposing the material to a high temperature and cooling it down to room temperature to carry out the testing. Testing after cooling gives the residual values corresponding to the post-fire performance of concrete.

### 2.3. Heat Treatment of Concrete Samples

Concrete specimens were subjected to a slow heating regime. The heating rate was 1 °C/min, starting from room temperature until reaching the critical temperatures (CTs). The CTs were held constant for 2 h to allow the homogenization of all specimens and subsequently cooled at a rate lower than 1 °C/min inside a Nabertherm electric furnace. The critical temperatures were set at 350, 550, and 750 °C. 

The critical temperatures were chosen according to the main chemical transformations in concrete exposed to high temperatures. The main chemical transformations are summarized as follows (see Table 3): when concrete is exposed to high temperatures, around 100 °C, a mass loss occurs, associated with the evaporation of the water contained in the capillary pores and the CSH gel pores [72]. Ettringite, considered one of the most thermally unstable hydrates that composes the cement, decomposes at temperatures between 110 °C and 150 °C [83,84]. At temperatures between 100 °C and 200 °C, the adsorbed water bonds in the micropores are destroyed, and the CSH gel chain starts to decompose [84,85,86]. In the temperature range of 200 °C to 350 °C, the CSH gel is considered to decompose, forming unstable intermediate structures [83,87]. Portlandite decomposes at around 450 °C, forming CaO and H_2_O [66,68,85]. Aggregates that are considered thermally more stable compared to cement paste decompose at around 575 °C when they are siliceous in nature and around 700 °C when they are limestone [54]. At around 1200 °C, the dehydrated cement paste and aggregates begin to melt [72,84].

### 2.4. Instrumental Methods

Many concrete specialists proposed that the change in properties at high temperatures is related to the chemical release of water (mass loss) in concretes. The weight loss of concretes at high temperatures is easily monitored by a gravimetric technique, i.e., by recording weight loss over temperature. The weight loss highly depends on the compositions (cement type and contents), hydration degree (curing temperature, humidity, and age), and the initial moisture states (saturated, air-dried, or oven-dried). In this study, the physical and chemical changes in concrete at high temperatures were evaluated by the weight loss in the concrete specimens at 350, 550, and 750 °C. The change in the concrete weight was evaluated by measuring the mass of samples using a precision balance (Mettler Toledo PM4800 DeltaRange Balance, Sydney, Australia) with an accuracy of 0.01 g. The weight loss (Wl) is expressed as a percentage of the original weight before heating (wi) to the weight after exposure to the different critical temperatures (ws) by using Wl = (wi − ws/wi) × 100.

The ultrasonic pulse velocity test (UPV) may be considered one of the most promising methods for concrete structure evaluation. The UPV is used as nondestructive testing that makes possible an examination of the material homogeneity and estimates the mechanical properties, the compressive strength, and the modulus of elasticity. The UPV results may be used for permeability, diagnosis, prognosis, and quality control. Based on ultrasonic pulse velocity measurements carried out in ordinary concrete by Feldman, Leslie, and Chessman, the concrete quality was classified as follows [88]: excellent for UPV > 4500 m/s, good for UPV between 3600 to 4500 m/s, questionable for UPV between 3000 to 3600 m/s, poor for UPV between 2100 to 3000 m/s, and very poor for UPV ≤ 2100 m/s. The samples were examined for physical deterioration by the ultrasonic pulse velocity technique (UPV) using a portable ultrasonic non-destructive digital indicator tester according to UNE-EN 12504-4 (PUNDIT PL-200, Proceq SA, Switzerland). The principle of the UPV test involves sending a wave pulse into the concrete sample, measuring the speed and the travel time in which the pulse propagates through the concrete. The UPV tests were carried out on the cylindrical specimens, reporting the average of more than five samples as the result. The test was accomplished after specimens were subjected to high temperatures to obtain the residual UPV.

The crystalline phases were identified by the X-ray diffraction powder technique (XRD, Bruker D8-ADVANCE, Bruker AXS GmbH, Karlsruhe Germany). The scans were performed in the 2θ range from 10 to 60° with a step scan of 0.016° and 60 s per step in a continuous mode.

The decomposition of concrete phases was carried out, analyzing the responses from 22 °C to 1000 °C in thermogravimetric equipment (TA Instrument model SDT Q600, New Castle, DE, USA). Samples of 20–30 mg were placed in an alumina crucible and heated at 10 °C/min in a nitrogen atmosphere (with a flow rate of 100 mL min^−1^), with alumina as reference material. 

One of the first assessments of concrete affected by fire or subjected to high temperatures is its physical appearance. Although this may not provide trusty structural information about the distortion suffered by the concrete, it can give an instant impression of the failure tendency of the concrete. Surface damage (cracking and color change) was evaluated by surface inspection of the concrete samples.

The effect of high temperatures on the microstructure of concrete specimens was studied by scanning electron microscopy in the backscattered electron modality (SEM, S-4800 Scanning Electron Microscopy, Hitachi, Japan and Bruker X-Flash Detector 5030, Bruker Co., Billerica, MA, USA).

## 3. Results and Discussion

### 3.1. Physical Properties

Table 4 shows the physical properties of concrete specimens. All physical property data represent an average of 15 concrete specimens for each batch (repetitively).

As observed, C-SF30 concrete developed the higher compressive strength among all experimental concretes at 28 and 365 days. The strength improvement of C-SF30 compared with the C100 concrete was 44.72% and 30.58% at 28 and 365 days, respectively. Meanwhile, C-GNSW30 reaches a higher compressive strength at 28 and 365 days among concretes with GNSW contents. The strength improvement of C-GNSW20 compared with the C100 concrete was 10.37% and 6.64% at 28 and 365 days, respectively. Meanwhile, the strength improvement of C-GNSW30 compared with the C100 concrete was 22.57% and 17.82% at 28 and 365 days, respectively. All experimental concrete reached higher strength than the CPC concrete.

On the other hand, the open porosity values reported for experimental concretes are very similar (around 21%) but slightly higher than CPC concrete. 

It is well known that including geothermal silica often decreases the workability of concrete. GNSW is very fine and has a high surface area, which increases the water demand to achieve a similar consistency compared to concrete without it. Superplasticizers or high-range water reducers are commonly used to counteract the decreased workability. These additives help to improve the flow and ease of placement of the concrete without compromising its strength or durability. 

When evaluating the workability of concrete containing geothermal silica, the values reported can vary based on factors such as the type and amount of GNSW used, the specific mix design, and any additional additives or adjustments made. The workability values obtained for experimental concrete are (i) C100, 150 mm (6 inches), (ii) C-GNSW20, 90 mm (3.5 inches), (iii) C-GNSW30, 75 mm (3 inches), and C-SF30, 75 mm (3 inches). In the literature, the degrees of workability that are well recognized are very low (0–25 mm), low (25–50 mm), medium (50–100 mm), and high (100–175 mm).

### 3.2. Weight Loss

**Figure 2:**
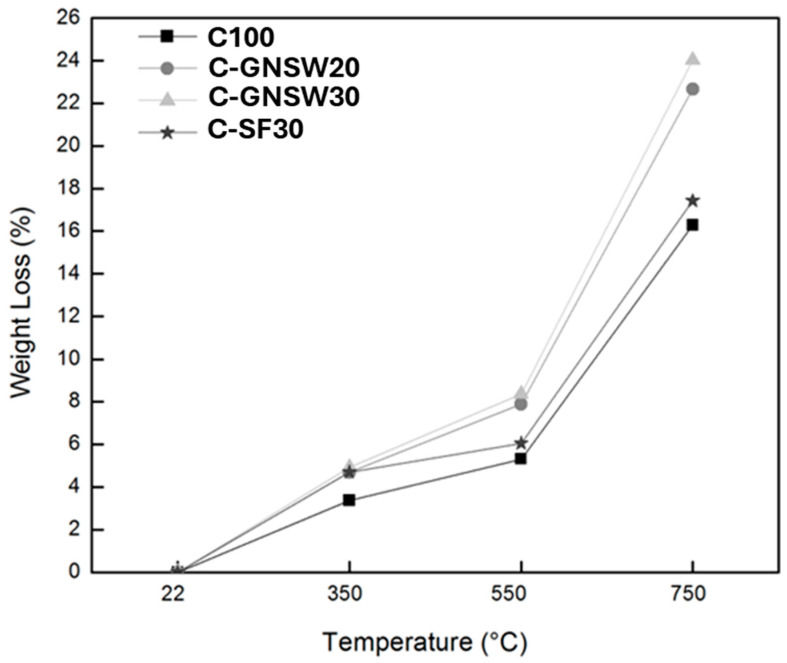
Weight losses of the concrete specimens.

Figure 2 shows the weight loss in the concrete specimens due to exposure to high temperatures. For all specimens, the percentages in weight loss increase with increasing temperature. As known, the increase in temperature in the concrete structure mainly leads to water vapor release, mainly from free water up to 100 °C and at higher temperatures, calcium aluminate hydration and C-S-H gel dehydration up to 350 °C, and calcium hydroxide around 450 °C [23]. The weight loss of the specimens exposed to the critical temperature of 350 °C was low, that is, 3.36%, 4.69%, 4.91%, and 4.7% for C100, C-GNW20, C-GNW30, and C-SF30 specimens, respectively. The weight loss at 350 °C may be attributed to free water evaporation, a slow capillary water loss, and a physically bound C-S-H water evaporation at temperatures up to 300 °C [77,89]. The weight loss of the specimens gradually increased when they were exposed to 550 °C, that is, 5.31%, 7.89%, 8.36%, and 6.04% for C100, C-GNW20, C-GNW30, and C-SF30 specimens, respectively. At 550 °C, the weight loss of the samples may be related to the decomposition of CH as well as some chemically bonded water losses from C-S-H gels. Some authors suggested that cement paste loses its bending properties at temperatures above 500 °C due to the thermal decomposition of constituents of hydration products [70,89,90]. Finally, the weight loss of all samples increased sharply when the temperature reached 750 °C, that is, 16.28%, 22.66%, 24.03%, and 17.43% for C100, C-GNW20, C-GNW30, and C-SF30 specimens, respectively. At temperatures ranging from 600 to 800 °C, the weight loss in concrete may be attributed to the decarbonization of coarse aggregates (limestone-CaCO_3_) [23].

In this study, the weight losses were higher for the specimens with partial replacements of GNSW and SF than the reference specimen (C100). As observed, at 350 °C, the weight loss of samples C-GNSW20, C-GNSW30, and C-SF30 was very similar. At 550 °C, the weight loss was higher for the specimen with GNSW addition, which can be attributed to the pozzolanic activity presented by the GNSW, which increases the amounts of CSH gel, leading to a much denser cementitious matrix, causing a higher weight loss compared to the reference specimens. This behavior agrees with previous reports about more C-S-H formation with mineral additions [77,91,92]. Meanwhile, when the specimens were exposed to 750 °C, the weight loss was higher for specimens with GNSW addition than those made only with CPC and SF additions. The weight loss of the specimen with SF additions almost resembles the reference specimen (100% CPC). Finally, the weight loss difference between C-GNSW20 and C-GNSW30 was too close at all critical temperatures. 

The weight loss percentages found in this research work are comparable to those values found by Morteza in his study. Morteza et al. studied the effect of high temperatures on high-strength concrete modified with silica nanoparticles and SF, where at 400 °C, the percentages of weight loss vary between 3 and 4% and between 12 and 18% at 800 °C [31].

### 3.3. Ultrasonic Pulse Velocity (UPV)

**Figure 3:**
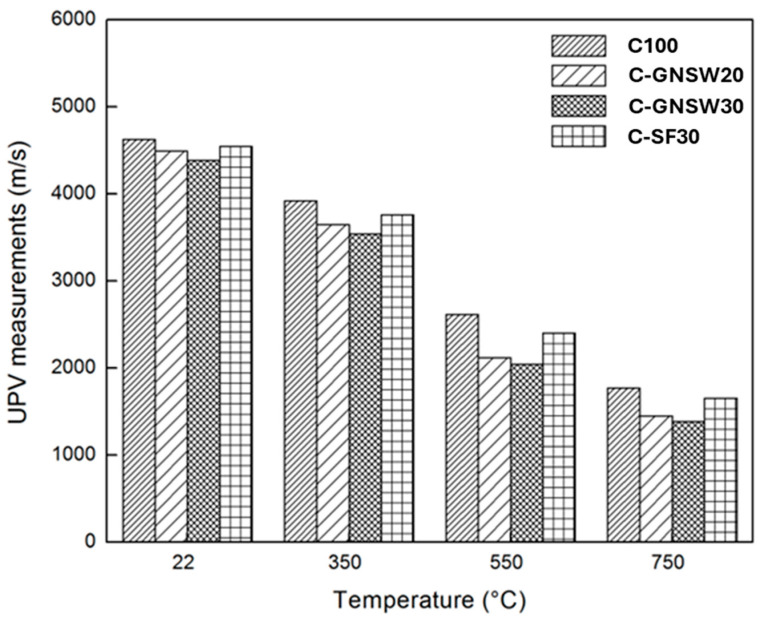
Residual ultrasonic pulse velocity of the concrete specimens.

Figure 3 shows the measurements of the residual UPV obtained in all concrete specimens at different critical temperatures. It can be observed as the temperature increases, the UPV decreases. This behavior is due to deterioration in the microstructure of the concrete samples, mainly because of pore size increase and the microcracking of dehydrated cement paste generated by exposure to high temperatures [75,79]. At room temperature, the UPV measurements suggest excellent quality in all samples. At 350 °C, the UPV measurements showed reductions in all the specimens; nevertheless, they were found in the range to be considered as good-quality specimens. On the other hand, at 550 and 750 °C, the reductions in the UPV measurements of all the samples allow them to be considered as specimens of questionable and poor quality, respectively. At temperatures close to 300 °C, the loss of physically absorbed free water is probably the cause of this decrease in the UPV since it generates the shrinkage of cement paste and microcracking as well as pore growth. At temperatures above 450 °C, the reduction in the UPV is mainly due to the degradation of the C-S-H gel and CH in plain OPC concrete. The poor quality shown by all concrete specimens when subjected to 750 °C is attributed to the complete degradation of the C-S-H gel and decarbonization of the coarse aggregate used, which considerably increased the number of cracks and porosity in the concrete. Therefore, the transmission speed of sound waves through the specimen decreases in these cases at high temperatures [93,94].

The reduction in the UPV was higher in all cases for concrete specimens with partial replacements (GNSW and SF) compared to the reference specimen (C100). GNSW and SF concrete specimens show more compact microstructures due to the pozzolanic activity and the filler effect of both supplementary cementitious materials. In the absence of adequate permeability, these factors can cause a buildup of vapor pressure formed by the evaporation of chemically and physically bound water inside the concrete, trying to find a way to release it, which leads to crack formation. In addition, it promoted higher porosity due to the decomposition of C-S-H gel, which is more abundant in these concrete specimens [68,69]. Therefore, superior damage is observed in the microstructure of specimens with partial replacement of GNSW and SF compared to the reference sample at different critical temperatures [91,95,96].

### 3.4. X-ray Diffraction (XRD)

**Figure 4:**
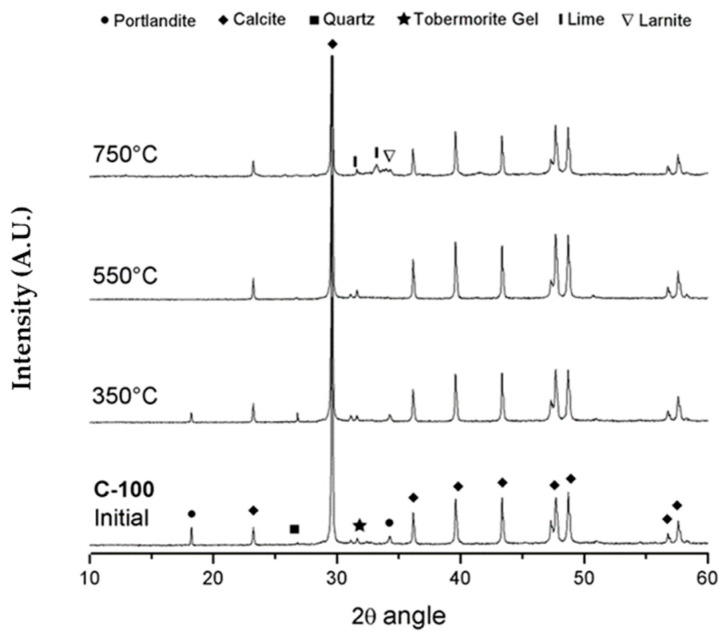
Diffractogram of C100 concrete before and after exposure to critical temperatures.

**Figure 5:**
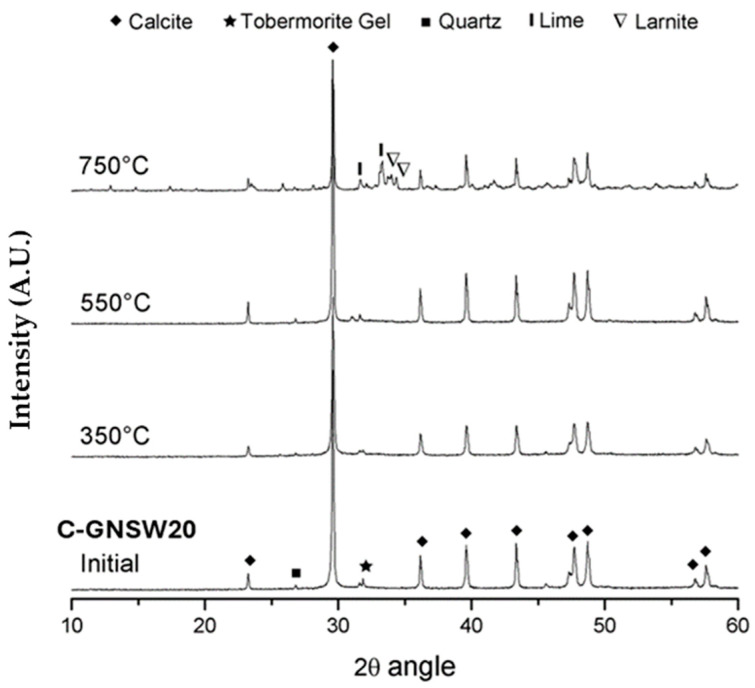
Diffractogram of C–GNSW20 specimen before and after exposure to critical temperatures.

Figure 4 and Figure 5 show the results of XRD for the reference specimen and the C-GNSW20 specimen before and after being subjected to different critical temperatures, respectively. The typical reflections associated with crystalline phases such as portlandite, calcite, quartz, larnite, lime, and dolomite were detected and identified, while ettringite was not detected in the specimens. The calcium silicate hydrated cannot be directly detected by this technique due to its amorphous nature. In the reference specimen, it is possible to observe how the peak intensity corresponding to the portlandite decreases after the exposition to the critical temperature of 350 °C. The portlandite was not detected in the diffractogram corresponding to 550 °C, while the peak intensity corresponding to calcite and the diffraction lines of C-S-H gel increase at this critical temperature. The free CaO formed after the decomposition of the portlandite can react with the CO_2_ from the inside of the furnace to form CaCO_3_, which explains the increase in the intensity of the peaks of calcite in the specimen subjected to 550 °C [87,97]. The peak intensity corresponding to the calcite decreases as the C-S-H gel is transformed into similar thermally stable minerals (mainly β-C_2_S and C_3_S) after exposure to the critical temperature of 750 °C [98]. 

In the C-GNSW20 specimen, the diffractogram at 22 °C allows us to observe that the portlandite was consumed. This behavior indicates the high pozzolanic activity of the geothermal nano-SiO_2_ waste to react with calcium hydroxide and generate superior amounts of hydrated products, mainly the C-S-H gel. The diffractogram at 22 °C and 350 °C did not show significant changes in the peaks of both concrete specimens, suggesting that the detected crystalline phases did not show transformations at this critical temperature. At 550 °C, an increase in the intensity of calcite peaks can be observed, as well as a subsequent decrease in the intensity when the critical temperature reaches 750 °C. The decrease in the intensity of calcite peaks at the temperature of 750 °C suggests the decomposition of it.

### 3.5. Thermal Analysis (TG-DTA)

**Figure 6:**
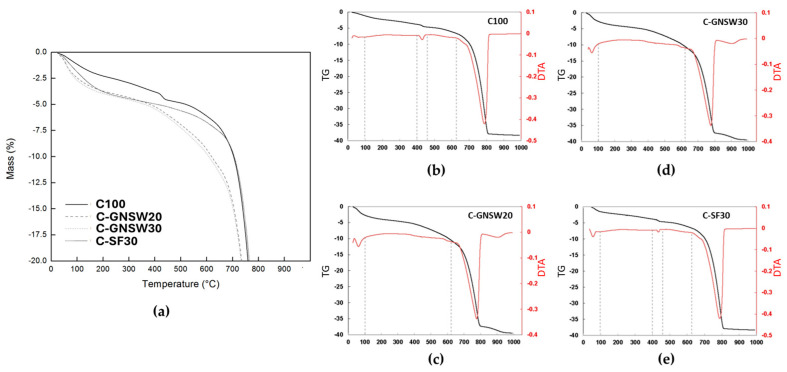
TG−DTA diagram of the initial concrete specimens. (**a**) TG diagram of the initial concrete specimens, (**b**) C100 TG−DTA diagram, (**c**) C−GNSW20 TG−DTA diagram, (**d**) C−GNSW30 TG−DTA diagram, (**e**) C−SF30 TG−DTA diagram.

Figure 6 shows the thermogravimetric analysis results represented by the mass variation of the initial concrete specimens from 22 °C to 1000 °C. As shown in the TG curves, four main steps represent instantaneous mass losses concerning temperature. The first step is observed in the range of 22 °C to 150 °C, corresponding to the temperature range in which the moisture (free water) of concrete specimens is evaporated. The second step is observed in the range of 150 °C to 400 °C, where the mass loss is mainly related to the loss of chemically bound water from the decomposition of the C-S-H gel. In this temperature range, the behavior of concretes with partial replacements of Portland cement by GNSW or SF is very similar, which agrees with the results obtained from the weight loss and the UPV measurements at the critical temperature of 350 °C. The third step is observed between 400 and 550 °C, corresponding to the water loss from the portlandite only observed in the reference specimen (C100). In this temperature range, the mass loss is more pronounced for specimens with GNSW replacements compared to the specimen with SF replacement, which can be attributed to the difference in particle size between the geothermal nano-SiO_2_ waste and the silica fume. This pronounced mass loss may be attributed to the fact that the silica nanoparticles incorporated into the cementitious matrix acted as a nucleation point that reacts actively with the portlandite in the hydration reactions, producing higher amounts of C-S-H gel. The C-S-H gel begins to disintegrate with other hydration products in this temperature range (400 °C to 670 °C) just right mentioned. Based on the TG curves shown by the C-GNSW20 and C-GNSW30 specimens, temperatures higher than 400 °C can be considered harmful because the main hydration products begin to disintegrate, so that the adhesion properties of the cement paste are lost, threatening the stability of the concrete structure. The last step of higher mass loss is observed above 670 °C, related to the decarbonization of the coarse aggregates, being a component of the concrete specimens [89,90,97,99,100].

TG-DTA analysis (Figure 6b–e) indicated that the C-GNSW20 and C-GNSW30 specimens showed similar behavior to the critical temperature exposure. Meanwhile, the silica fume as a partial replacement of Portland cement has already been widely studied in mortars and concretes at high temperatures, showing a similar behavior as C100 concrete. Henceforth, only the C-GNSW20 specimen is compared with the C100 reference specimen [72].

**Figure 7:**
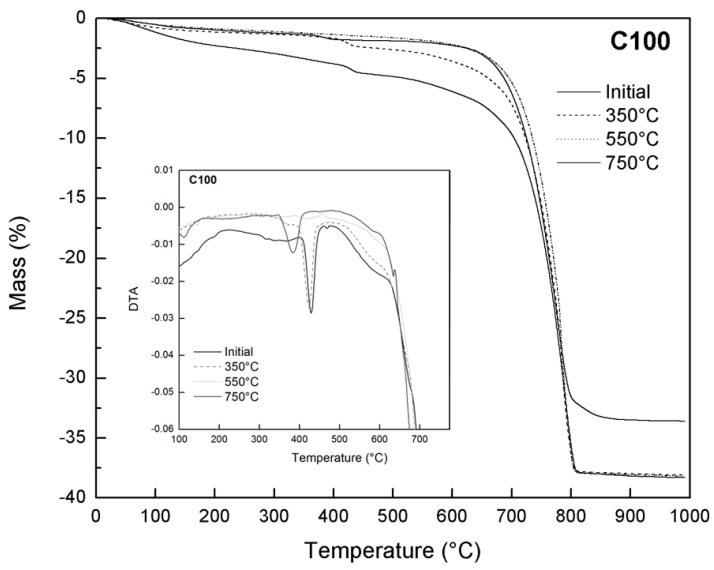
TG of C100 concrete specimen at different critical temperatures. DTA is shown in inset.

Figure 7 shows the thermogravimetric analysis (TG) and the differential thermal analysis (DTA) of the C100 reference specimen before and after being subjected to the different critical temperatures. The DTA analysis shows that the peak corresponding to the portlandite (400–450 °C) is identified in the specimen at 22 °C and in the specimen subjected to 350 °C. This peak was not detected when the specimen was exposed to 550 °C. However, the peak is once again appreciated after cooling from 750 °C but shifted to the left of the curve [24]. This peak displacement suggests that the portlandite formation was accomplished during the cooling step caused by rehydration, i.e., atmosphere moisture capture. In addition, it is proposed that this new portlandite is less crystalline [83]. Although the portlandite has decomposed to form CaO and H_2_O at 550 °C, no new portlandite formation has been detected during the cooling. On the other hand, during the cooling of the specimen heated to 750 °C, the formation of portlandite was observed. This behavior suggests that more severe heating is required for the new portlandite formation. The rehydration of portlandite can occur when CaO reacts with water during the fire extinguishing process, leading to a significant volumetric change. One of the reasons for using the SCMs is related to their pozzolanic activity, leading to a higher portlandite consumption. 

**Figure 8:**
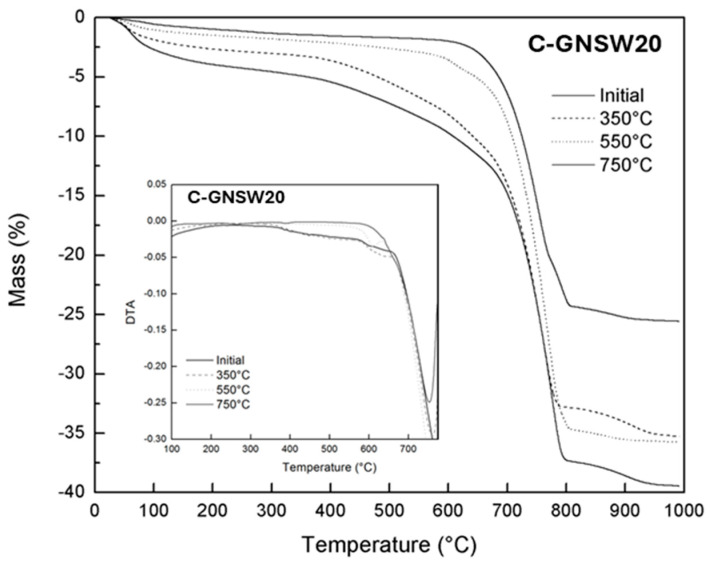
TG of C−GNSW20 concrete specimen at different critical temperatures. DTA is shown in inset.

This event is appreciated in the DTA illustrated in Figure 8 (a region corresponding to a temperature around 450 °C). The absence of CH can contribute to less cracking due to the diminished volumetric changes caused by the transformation of Ca(OH)_2_ into CaO and H_2_O [73].

### 3.6. Surface Inspection and Scanning Electron Microscopy (SEM)

**Figure 9:**
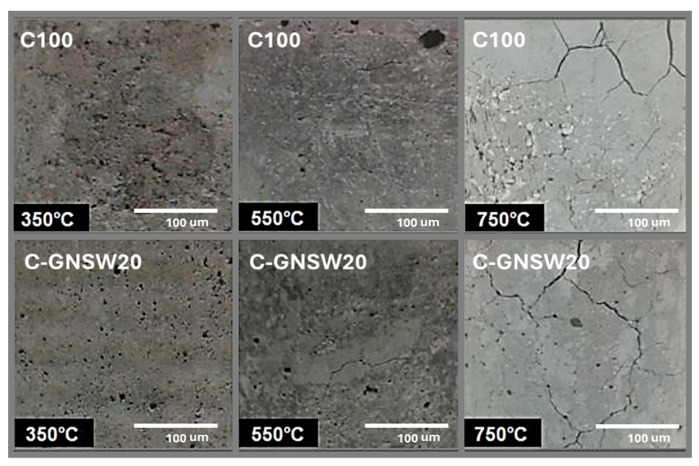
Surface damage of the C100 and C–GNSW20 specimens at different critical temperatures.

Figure 9 shows the surface damage suffered by C100 and C-GNSW20 specimens after being subjected to the different critical temperatures. From this inspection analysis, it is possible to observe that the incidence of cracks on the surface of both specimens was minimal when they were exposed to 350 and 550 °C. Furthermore, at 750 °C, the cracking and color change on the specimen’s surface. This phenomenon agrees with Arioz et al. research wherein for concrete made with OPC and exposed to temperatures up to 1200 °C, cracking and significant color changes occur at temperatures above 600 °C. In addition, Ali et al. found no significative color changes in reinforced concrete subjected to temperatures below 400 °C. Meanwhile, there was a slight change in color between 400 and 600 °C. Finally, the color change was remarkable at temperatures close to 800 °C [66,101].

The high water vapor pressure, decomposition of the hydration products, and thermal incompatibility between the cement paste and aggregates are considered the main factors that significantly affect the crack propagation into a specimen subjected to high temperature [94,95,98].

**Figure 10:**
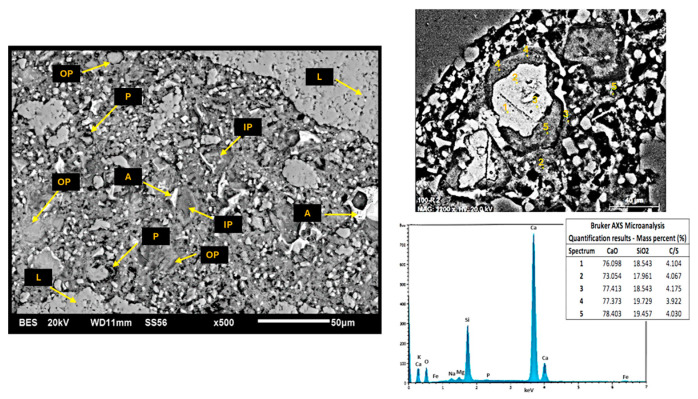
SEM micrograph of initial C100 concrete specimen. L: limestone aggregate, A: anhydrous cement, IP: inner product, OP: outer product, P: porosity.

**Figure 11:**
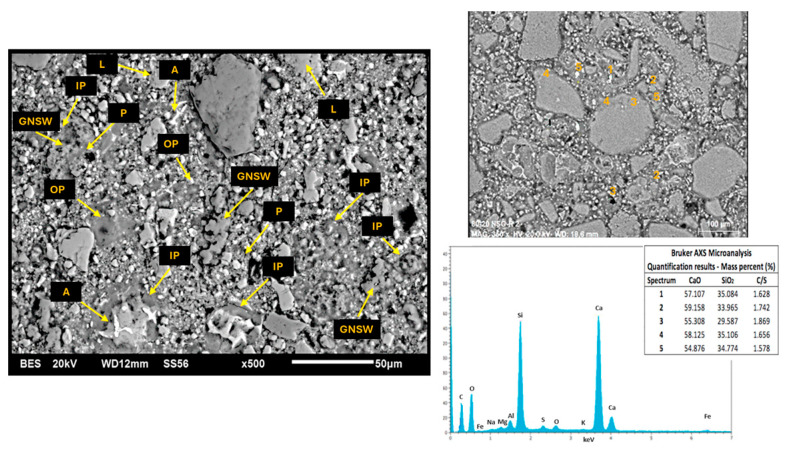
SEM micrograph of initial C–GNSW20 concrete specimen. L: limestone aggregate, A: anhydrous cement, IP: inner product, OP: outer product, GNSW: geothermal nano-SiO_2_ waste, P: porosity.

Figure 10 and Figure 11 show the SEM micrographs corresponding to the C100 and C-GNSW20 (at 22 °C) concrete microstructures. Both microstructures show similar characteristics, such as a well-distributed cementitious matrix with partially hydrated cement grains, inner and outer products, and some dispersed porosity. However, the C-GNSW20 microstructure is denser compared to the C100 microstructure. As aforementioned, the silica nanoparticles react with the calcium hydroxide to produce an additional C-S-H gel. This phenomenon promotes densification of the cementitious matrix, reducing the porosity and permeability, which may explain the higher values in compressive strength (29.19 MPa) compared to the reference specimen (27.37 MPa). This argument was proved by EDS punctual analysis, where a Ca/Si ratio (mass %) of 4.01 and 1.79 was found for C100 and C-GNSW20 specimens, respectively [97].

**Figure 12:**
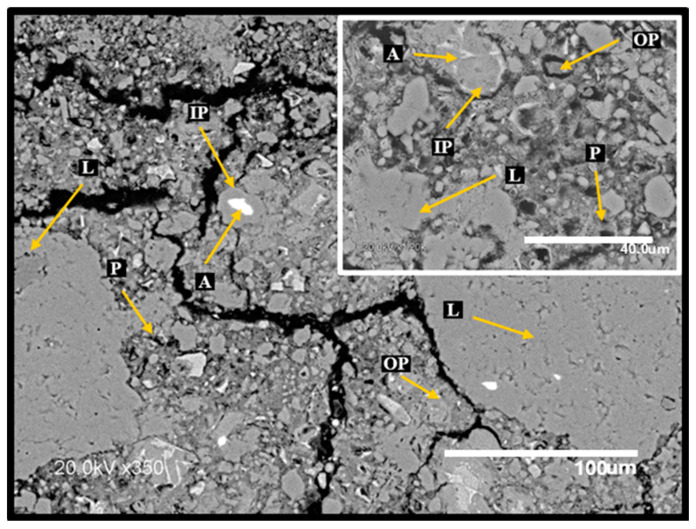
SEM micrograph of C100 specimen subjected at 750 °C. L: limestone aggregate, A: anhydrous cement, IP: inner product, OP: outer product, P: porosity.

**Figure 13:**
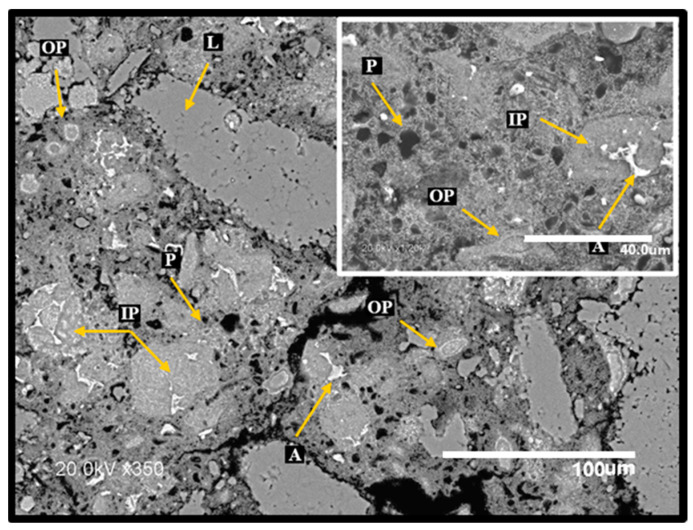
SEM micrograph and magnification of C–GNSW20 specimen subjected at 750 °C. L: limestone aggregate, A: anhydrous cement, IP: inner product, OP: outer product, P: porosity.

Figure 12 and Figure 13 show the SEM microstructure of C100 and C-GNW20 specimens after being subjected to 750 °C. Through these SEM images, it is possible to observe the microstructure damage of both specimens. The appearance of cracks in the aggregate–cement paste interface is evident. Also, a mismatch between the cement pastes and the aggregates due to the shrinkage of the cement paste with the increase in temperature can be observed. At the critical temperature of 750 °C, the decomposition of the C-S-H gel is inevitable. This issue causes the loss of bonding properties between the concrete components, hurting the mechanical and durability properties. As higher amounts of C-S-H gel were generated in the C-GNSW20 specimen, superior damage in the microstructure was observed. This behavior is attributed to the decomposition of this gel and other hydration products. A higher hydrate decomposition was reflected in the C-GNSW20 specimen than in the reference specimen, where microcracking and coarsening of the pore structure can be distinguished at higher magnification (see Figure 12). High porosity and air voids in the microstructure of the C-GNSW20 specimen explain the low values of the UPV obtained at this critical temperature [100,102].

## 4. Conclusions

This study evaluated the effect of replacing Portland cement with geothermal nano-SiO_2_ waste (GNSW) at 20% and 30% on the durability of concrete subjected to high temperatures. The key findings are as follows:

Weight Loss: Concrete with GNSW and SF showed minimal weight loss at 350 °C, which increased at 550 °C and sharply at 750 °C. There was a negligible difference between 20% and 30% GNSW replacements.

UPV Results: All specimens maintained good quality at 350 °C, but concretes with GNSW exhibited lower UPV at 550 °C and 750 °C, indicating more damage due to their denser microstructure.

Thermal Behavior: TG curves indicated similar thermal stability up to 400 °C, beyond which GNSW-replaced concretes showed greater mass loss.

DTA Results: The formation of Ca(OH)_2_ and CaCO_3_ was observed at 750 °C, suggesting reactions during the cooling stage.

Microstructure: Micrographs revealed a denser structure in C-GNSW20, attributed to increased C-S-H gel formation and a filler effect, though higher magnification showed microcracks and pore coarsening due to C-S-H decomposition.

In conclusion, GNSW is a viable supplementary cementitious material, enhancing thermal properties up to 400 °C while offering environmental benefits by reducing industrial waste.

## Figures and Tables

**Table 1:** Chemical composition and physical properties of raw materials.

Compound	Compound Portland Cement (CPC)	Silica Fume (SF)	Geothermal Nano-Silica Waste (GNSW)
	(wt.%)		
SiO_2_	18.51	95.89	98.36
Al_2_O_3_	4.46	0.42	0.09
Fe_2_O_3_	2.61	1.22	0.04
CaO	67.45	0.61	0.45
MgO	1.27	0.42	-
SO_3_	3.26	0.45	0.03
Na_2_O	0.36	0.17	0.32
K_2_O	0.87	0.82	0.23
Cl^−^	-	-	0.06
LOI	0.79	0.29	0.31
Density (g/cm^3^)	3.03	2.20	2.04
BET Area (m^2^/g)	0.88	24.66	8.56

**Table 2:** Designation and mixture proportion of concrete specimens.

Materials	Sample Code Partial Replacements (wt.%)			
	C100	C-GNSW20	C-GNSW30	C-SF30
Portland cement	14.86	11.89	10.40	10.40
SCM (GNSW or SF)	0	2.97	4.46	4.46
Coarse aggregate	42.46	42.46	42.46	42.46
Fine aggregate	35.03	35.03	35.03	35.03
Water	7.43	7.43	7.43	7.43
Superplasticizer	0.22	0.22	0.22	0.22
Total	100	100	100	100

**Table 3:** Main chemical transformations in concrete exposed to different temperatures.

Temperature Range	Chemical Transformation in Concrete
20–80 °C	Water slow elimination contained in the capillary pores
80–90 °C	Ettringite (Aft) and monsulfate (AFm) decomposition
80–100 °C	Water loss contained in the capillary pores
100–200 °C	Chemical water elimination (absorbed and interlaminar)
200–350 °C	CSH gel decomposition into αC_2_SH
400–450 °C	(CaOH)_2_ decomposition into CaO and H_2_O
570–600 °C	αSiO_2_ transformation into βSiO_2_
650–800 °C	CaCO_3_ decomposition into CaO and CO_2_
800–1200 °C	Dehydrated phases melting
>1200 °C	Aggregates melting

**Table 4:** Physical properties of concrete specimens.

Physical Properties	Sample Code Partial Replacements (wt.%)			
	C100	C-GNSW20	C-GNSW30	C-SF30
Workabilty (mm)	150	90	75	75
Open porosity (%)	18.46	21.47	21.76	21.54
Compressive strength at 28 days (MPa)	23.03	25.42	28.23	33.33
Compressive strength at 365 days (MPa)	27.37	29.19	32.25	35.74

## Data Availability

Data are contained within the article.
